# Advances in T Cells Based on Inflammation in Metabolic Diseases

**DOI:** 10.3390/cells11223554

**Published:** 2022-11-10

**Authors:** Wenlu Yu, Chunxiu Li, Deju Zhang, Zhangwang Li, Panpan Xia, Xiao Liu, Xia Cai, Pingping Yang, Jitao Ling, Jing Zhang, Meiying Zhang, Peng Yu

**Affiliations:** 1 Department of Metabolism and Endocrinology, The Second Affiliated Hospital of Nanchang University, Nanchang 330000, China; 2 School of Ophthalmology and Optometry, Nanchang University, Nanchang 330000, China; 3 Food and Nutritional Sciences, School of Biological Sciences, The University of Hong Kong, Pokfulam Road, Hong Kong 999077, China; 4 The Second Clinical Medical College, Nanchang University, Nanchang 330000, China; 5 Department of Cardiology, Sun Yat-sen Memorial Hospital of Sun Yat-sen University, Guangzhou 510000, China; 6 Department of Anesthesiology, The Second Affiliated Hospital of Nanchang University, Nanchang 330000, China

**Keywords:** metabolic diseases, CD4+ T cells, CD8+ T cells, inflammation, signaling pathway

## Abstract

With the increasing incidence of metabolic diseases year by year and their impact on the incidence of cardiovascular diseases, metabolic diseases have attracted great attention as a major health care problem, but there is still no effective treatment. Oxidative stress and inflammation are the main mechanisms leading to metabolic diseases. T cells are involved in the inflammatory response, which can also regulate the development of metabolic diseases, CD4+ T cells and CD8+ T cells are mainly responsible for the role. Th1 and Th17 differentiated from CD4+ T promote inflammation, while Th2 and Treg inhibit inflammation. CD8+ T cells also contribute to inflammation. The severity and duration of inflammatory reactions can also lead to different degrees of progression of metabolic diseases. Moreover, mTOR, PI3K-Akt, and AMPK signaling pathways play unique roles in the regulation of T cells, which provide a new direction for the treatment of metabolic diseases in the future. In this review, we will elaborate on the role of T cells in regulating inflammation in various metabolic diseases, the signaling pathways that regulate T cells in metabolic diseases, and the latest research progress.

## 1. Introduction

Metabolic disease is a kind of multi-system abnormal disease which is manifested by diseases or disorders that disrupt normal metabolism, including hyperglycemia, dyslipidemia, hypertension, obesity, and insulin resistance, and leads to a dramatic increase in the occurrence of cardiovascular diseases such as acute myocardial infarction and stroke [1]. Compared to those without metabolic diseases, individuals with them are twice as likely to suffer from cardiovascular disease [2]. In 2018, several studies showed that nearly one-third of Americans suffered from metabolic diseases. Meanwhile, the prevalence of obesity doubled in 73 countries and a significant increase has been observed in most other countries since 1980, which can also promote the emergence of metabolic diseases [3]. Furthermore, the incidence of metabolic diseases is on the rise globally due to the lack of effective treatments [4]. A number of factors are associated with metabolic dysfunction, such as cells, tissues, organs, inflammatory signaling cascades, and humoral factors [1]. It is now widely acknowledged that oxidative stress along with chronic inflammation is positively related to the initiation and development of metabolic diseases [5,6]. Dysregulation of gut microbiota and pro-opio-melano-cortin neurons are also closely associated with the incidence of metabolic diseases and dysregulation of autophagy [7,8,9]. Inflammation or inflammatory response is a series of intense reactions produced by human tissues and organs in response to the destructive factors of pathogens. Moderate inflammation promotes cell survival, whereas long-term chronic or excessive inflammatory response often leads to a series of diseases that are generally recognized as a fundamental pathological situation, when people are obese, inflammatory cells infiltrate and accumulate into adipose and other tissues [10]. Chronic inflammation, characterized by abnormal occurrences of cytokines, increases the development of acute reactants and other media, and is closely associated with obesity, insulin resistance, and type 2 diabetes (T2D) [11].

T cells play an indispensable role in inflammation and are directly involved in the development of metabolic diseases, for example: (1) During chronic inflammation, the balance of Th17/Treg is closely related to metabolic diseases [12]. (2) T cell phenotyping help stratify the obese and/or T2D patient and exhibit positive therapeutic and prognostic implications [13]. (3) T cell senescence is related to altered hepatic glucose homeostasis [14]. In this review, we will describe the effect on T cells in the regulation of inflammation in various metabolic diseases and the latest research progress.

## 2. Mechanism of Inflammatory Response and T Cell

### 2.1. T Cell

T lymphocytes originate from bone marrow, mature in the thymus, then differentiate into two major T cell lineages with different immune functions. A small fraction belongs to γδ-lineages, and migrate from the thymus to the epidermis, mucosa, and intestine, acting as the natural surveillance cells of the immune system, but most T cells belong to αβ-lineage [15]. Immature αβ-T cells in the thymus are also known as thymocytes at various stages of development, and each stage of cells has a different phenotype, from CD4− CD8− double-negative (DN) and CD4+ CD8+ double-positive, to CD8+ or CD4+ single-positive T cells [16]. T cells can express various surface membrane molecules, which participate in the antigen recognition, activation, proliferation, differentiation, and effector function of T cells. Among them, the T cell antigen receptor (TCR)-CD3 complex, the CD4, and the CD8 molecules are the most common surface membrane molecules and their functional roles are listed as follows [17]. TCR-CD3 is expressed on the surface of all T lymphocytes [18]. CD4 or CD8 are expressed on the surface of mature T cells and are divided into CD4+ T cells or CD8+ T cells [19]. Treg cells are a specific lineage of CD4+ T cells, which participate in maintaining immune homeostasis and restricting excessive immune responses, inhibiting the activation of primary T cells [12,20].

### 2.2. The Role of T Cells in Acute Inflammation

In the early phase of inflammation, cytokines such as IL-6 and tumor necrosis factor-α (TNF-α) are produced by T cells, as inflammatory stimuli, which can be recognized by pattern-recognition receptors. After that, inflammatory pathways including nuclear factor kappa-B (NF-κB) pathway, mitogen-activated protein kinase (MAPK) pathway, and Janus kinase signal transducer and activator of transcription (JAK-STAT) pathway are activated. In addition, the NF-κB pathway can regulate the biological processes of T cell-derived pro-inflammation factors such as IL-6, interferon-γ (IFN-γ), and granulocyte macrophage-colony stimulating factor (GM-CSF). However, acute inflammation is mainly regulated by innate immunity, and the recruitment of effector cells to infectious sites is the master ingredient of innate immunity. The γδ T cells could directly kill infectious cells through releasing cytotoxic granules and granulysin. Meanwhile, they can indirectly eliminate microbes by secreting pro-inflammation cytokines [21]. Most importantly, γδ T cells also produce IL-17 which are mainly secreted by Th17 cells. A study reported that IL-17 can promote the expression of chemokines and the recruitment of neutrophils and macrophages to the site of inflammatory tissue [22]. Numerous immune cells are recruited to injury tissue by inflammatory cytokines [23], which further promote pro-inflammatory environment and activate specific immunity. Under the induction of chemokines, naive T cells in the blood adhere to high endothelial venules, migrate along the venules in search of entry sites, and finally enter the lymph nodes. In the lymph nodes, T cells recognize antigens, activate and differentiate into mature T cells, then enter the blood to reach the inflammatory tissue [24]. CD4 + and CD8+ T cells are two major immune cells involved in inflammation, which play an indispensable role in the recognition and activation of T cells through antigen responses associated with major histocompatibility complex (MHC) class II molecules and MHC class I molecules, respectively [25]. Activated T cells secrete cytokines (TNF, IL-17, chemokines) which participate in recruiting macrophages and producing IFN-γ to activate macrophages, and also T lymphocyte subpopulations activate macrophages by producing different types of cytokines, then the activated macrophages stimulate T lymphocytes by antigen presentation and different cytokines (IL-12, IL-6, and IL-23) [26].

### 2.3. The Role of T Cells in Chronic Inflammation

**Figure 1:**
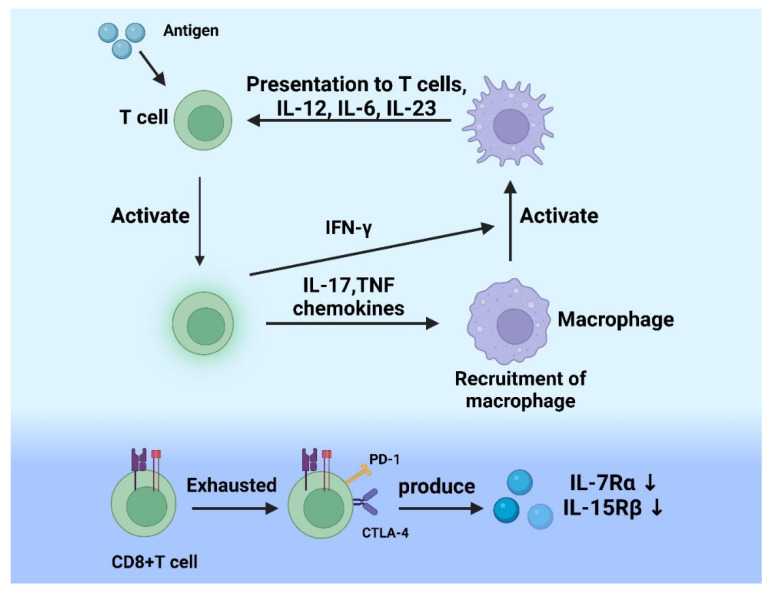
The relevant mechanisms of T cells.

Acute inflammatory state may switch to chronic state when immune cells are unable to eliminate pathogens [26]. Antigen-specific CD8+ T cells play a crucial role in controlling chronic infections. When acute inflammation occurs, CD8+ T cells differentiate into exhaustion state, and exhibit different functions compared to naive, effector, or memory CD8+ T cells [23,27,28,29,30]. During continuous stimulation of antigen, the exhausted CD8+ T express inhibitory receptors persistently, including programmed cell death protein-1 (PD-1) and cytotoxic T lymphocyte-associated antigen-4 (CTLA-4) [30,31,32,33]. Then the expression of inhibitory receptors results in the reduction of effector function and proliferative capacity [27]. Therefore, unlike memory CD8+ T cells, exhausted CD8+ T cells depend on antigenic stimulation to divide and proliferate [34]. Likewise, during chronic infections, the IL-18Rα is negatively regulated, and the responsiveness to some specific combined inflammatory cytokines (IL-12, IL-18, and IL-21) is decreased by CD8+ T cells [35]. The relevant mechanisms are summarized in Figure 1.

During acute inflammation, T cells are activated after T lymphocytes recognize the antigen. Activated T cells secrete cytokines (TNF, IL-17) which can participate in recruiting macrophages and produce IFN-γ to activate macrophages. The activated macrophages stimulate T lymphocytes by antigen presentation and different cytokines at the same time. During chronic inflammation, CD8+ T cells express inhibitory receptors such as PD-1 and CTLA-4, then exhausted, and the effector function and proliferative capacity reduce. TNF: tumor necrosis factor; IL-17: interleukin-17; PD-1: programmed cell death protein 1; CTLA-4: cytotoxic T lymphocyte-associated antigen-4. The figure was constructed with BioRender (https://biorender.com, accessed on 7 September 2022.).

## 3. The Role of Inflammatory Response in Metabolic Diseases

**Figure 2:**
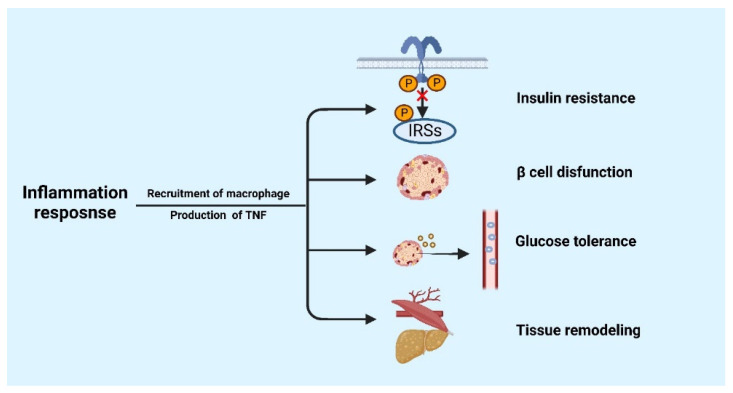
The role of inflammation response in metabolic diseases.

The immune system and the metabolic system work together to maintain the balance of metabolic homeostasis. The imbalance between these two systems can lead to chronic, low-grade systemic inflammation, which results in metabolic disorders [36]. The constantly rising levels of inflammatory mediators initiate the development of various chronic diseases such as obesity, T2DMellitus, atherosclerosis, non-alcoholic fatty liver disease, and gout [37,38]. Recently, several scholars have suggested that inflammatory response may develop insulin resistance and glucose intolerance, induce β-Cell dysfunction, promote tissue remodeling, and so on [39,40]. Figure 2 summarizes the effect of inflammation on metabolic diseases.

Studies have shown that obese mice developed insulin resistance in muscle and fat by blocking part of the NF-κB and Jun N-terminal kinase (JNK) pathways, which is attributed to the suppression of inflammatory signals [41,42]. Meanwhile, proinflammatory signaling molecules (TLR4), inflammasome instigates (NLRP3), and scaffolding proteins (STAMP2) can block the association between obesity and insulin resistance [43,44,45]. Proinflammatory cytokines act on metabolic diseases by facilitate recruiting macrophages and secreting TNF. Stephens and Pekala have reported that TNF has inhibitory effect on glucose transporter 4 (GLUT4) in adipocytes [46]. Another critical study conducted by Feinstein et al. illustrated that signaling cascades downstream of the insulin receptor can be inhibited by TNF [47]. Mahdi discovered that treatment of mice islets with pro-inflammatory factors such as IL-1β, IL-23, and IL-24 can significantly reduce glucose-stimulated insulin secretion, which implies β-Cell dysfunction [48,49,50].

Moreover, inflammation also promotes tissue remodeling. These responses involve stimulating angiogenesis to combat hypoxia, developing insulin resistance to protect adipocytes from lipids overaccumulation [40], and expanding adipose tissue (AT) to prevent ectopic fat accumulation in lipotoxic sites, such as liver and muscle [51].

Pro-inflammatory cytokines promote the recruitment the macrophage and secretion of TNF to act on metabolic diseases including insulin resistance, β cell dysfunction, impaired glucose tolerance, and tissue remodeling. TNF: tumor necrosis factor. The figure was constructed with BioRender (https://biorender.com, accessed on 7 September 2022).

## 4. The Function of T Cells in Regulating Inflammatory Response in Metabolic Diseases

### 4.1. CD4+ T Cells

**Figure 3:**
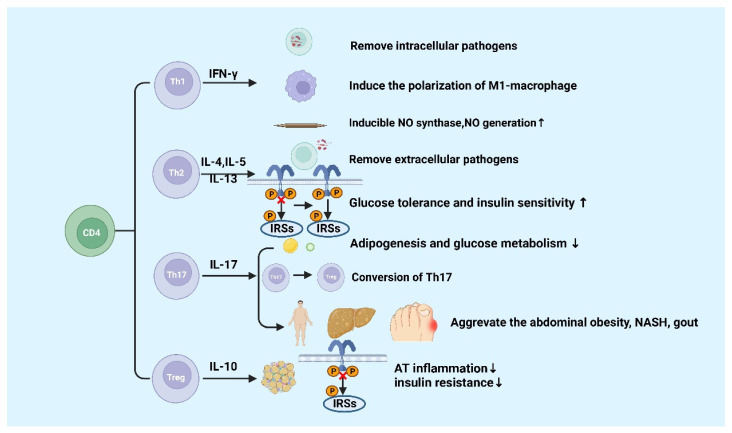
Functions of CD4+ T cells in metabolic diseases.

CD4+ T cells are important in metabolic diseases. Functionally, CD4+ T cells are also known as Th cells. Under the stimulation of different antigens and cytokines, CD4+ T cells can differentiate into four subsets, including Th1, Th2, Th17, and inducible regulatory T (iTreg) cells [52,53]. Th1 cells, which secret IFN-γ, exert the function of helper cellular immunity against intracellular pathogens and viruses, and contribute to the progression of chronic inflammatory diseases [54]. Th2 cells secrete cytokines such as IL-4, IL-5, and IL-13, which promote the proliferation and differentiation of B cells, assist humoral immunity, eliminate extracellular pathogens, and play a role in allergic inflammation [55]. Th17 cells can eliminate extracellular pathogens. Similar to Th1 cells, Th17 cells promote progression of inflammation [56]. Treg cells including iTreg and nTreg inhibit inflammatory response by secreting cytokines interculin-10 (IL-10) and transforming growth factor-β (TGF-β) [55]. Generally, Th1 and Th17 cells play a critical role in promoting pro-inflammatory responses, whereas Th2 and Treg cells are highly correlated with anti-inflammatory responses [57,58] (Figure 3).

IFN-γ is produced by Th1 cells. Studies showed that the level of IFN-γ is higher in high-fat diabetes (HFD)-induced obese mice and obese populations [59]. Furthermore, IFN-γ originating from Th1 promotes the conversion of macrophages to M1 macrophages, which will exacerbate AT inflammation, promote the production of chemokines and inflammatory cytokines such as TNF-α in AT [59,60,61]. In gouty inflammation, IFN-γ could synergistically act with monosodium urate (MSU) crystals to increase the expression of inducible NO synthase and NO production in macrophages, thereby causing pro-inflammatory immune responses [62]. Ricardo-Gonzalez et al. found that in mice that suffered from allergic inflammation with Th2 bias, the glucose tolerance and insulin sensitivity greatly improved [63]. Although the function of Th-related cytokines in inflammation response in metabolic diseases has been clearly defined, the mechanisms of Th1 and Th2 cells in metabolic diseases are not well understood [64].

IL-17 is mainly secreted by Th17 cells, which suppresses glucose metabolism and adipogenesis and postpones obesity progression in mice and humans [65]. A study of Ghannam reported that IL-17 deficiency would enhance diet-induced obesity (DIO) and change glucose homeostasis. Interestingly, it was previously reported that Th17 cells can adhere to MSCs during inflammation, which contributes to their transfer to a Treg-like phenotype [66]. However, further studies found that the number of Th17 cell in abdominal subcutaneous AT in insulin-resistant individuals with obesity are higher than those insulin-sensitive individuals with obesity [67]. It was reported that inflammatory Th17 cells increased during non-alcoholic steatohepatitis (NASH) in human and mouse disease models [68,69,70]. Additionally, IL-17 expression increases cytokine production in hepatocytes [12,71]. CD4+ T cells from DIO mice could secrete high levels of IL-17. The increased levels of IL-17 induce the production of IL-8, which can regulate various immune cell infiltration into AT in response to HFD [72]. Clinical studies have discovered that level of circulating IL-17 increases in patients with gout [73,74]. These findings suggested that Th17 cells and IL-17 might be the core factor leading to metabolic diseases, except for obesity [12].

Tregs exhibit an anti-inflammatory effect via IL-10 [12], which has been confirmed in many studies. The amount of visceral adipose tissue (VAT) Treg cells significantly reduces in HFD-fed obese mice [75], showing that HFD can destroy the immune system and trigger inflammation in mice. The dramatic reduction of Treg cells in VAT has been reported to be significant in adipose inflammation and insulin resistance [75,76]. Studies have found that the increase in adipose Treg cells plays a role in attenuating adipose inflammation and promoting insulin sensitivity in HFD-fed obese mice [77,78,79]. The transfer of Treg cells reduced the expression of TNF-α and the degree of HFD-induced hepatic inflammation [80]. However, a recent study showed Tregs deficiency inhibited the progression of NASH to hepatic cell carcinoma (HCC) in a mouse model of choline deficiency, HFD feeding, and diethylnitrosamine injection [81]. Increased pro-inflammatory T cell subgroups are recorded in the natural exhaustion of Tregs in both insulin-resistant mice and T2D patients [75,82,83]. In addition, Markus Feuerer and Yaron Ilan1 have also found that inflammation and insulin resistance in mice are protected by Tregs ex vivo or in vivo [75,78]. These results indicated that Tregs suppress T2D and promote metabolic health. These results indicated that Tregs inhibit inflammation and promote insulin resistance and may participate in the progression of metabolic diseases such as obesity, NASH, and T2D further. Finally, Hyperuricemia could improve spleen Th17 proportion as well as decrease Tregs proportion in mice, thus disrupting the Th17/Tregs functional balance is crucial in gout [84]. The specific molecular mechanisms of metabolic diseases about the different roles of Th17 and Treg cells are still poorly known and require continued investigation.

Naïve CD4 T cells differentiate into different subsets in various circumstances, including Th1, Th2, Th17, and Treg cells. Generally, Th1 and Th17 cells secrete pro-inflammatory cytokines, and Th2 and Treg cells produce anti-inflammatory cytokines. The figure was constructed with BioRender (https://biorender.com, accessed on 7 September 2022).

### 4.2. CD8+ T Cells

CD8+ T cells differentiate into cytotoxic T lymphocyte (CTL) after being stimulated by antigens. CTL kills target cells by secreting cytotoxic substances such as perforin and granzyme, and induces apoptosis through the Fas/FasL pathway [82]. It was reported that CD8+ T cells play a crucial role in chronic inflammation and metabolic disorders induced by metabolic disease [85]. It has been revealed that the population of CD8+ T cells increases in the AT of both DIO and gene-induced obese mice [59,86]. Additionally, the proportion of CD4+ to CD8+ T cells decreases in obese AT prior to infiltration of macrophages [87]. Notably, the CD8+ T cells also infiltrated in human AT [85]. Moreover, in CD8-deficient obese mice, the M1 macrophages and CD8+ T cells in AT significantly reduce, AT inflammation, glucose intolerance, and the insulin resistance also decrease [82].

CD8+ T cell infiltration increase in both obese humans and mice with nonalcoholic fatty liver disease (NAFLD) [88]. In mouse models, the consumption of CD8+ T cells reduces the liver damage in NASH, indicating that CD8+ T cells may directly promote disease development [89,90]. It is reported that compared to regular NASH mice, CD8+T cell-deficient mice are associated with higher hepatic insulin sensitivity, lower liver damage, and lower fibrosis [88,89,91]. There are several probable mechanisms about activated CD8+ T cells leading to NASH development. In the early transition from steatosis to NASH, type I interferons contribute to the activation of cytotoxic CD8+ T cells, resulting in increased production of pro-inflammatory cytokines such as IFN-γ and TNF-α [91]. During the establishment of NASH, the amount of liver tissue-resistant CXCR6+CD8T cells subsets increased, which could stimulate the role of self-attack to hepatic cells and instigate the disease development [92]. Importantly, during NASH, exhausted CD8 T cells would accumulate in liver tissues, which not only damages liver tissues but impairs immunosurveillance and promotes the conversion from NASH to hepatocellular cancer [93]. However, CD8+ tissue-resident memory T cells are also beneficial for murine NASH subsiding through inducing activated hepatic stellate cells (HSCs) to apoptosis [94]. Therefore, CD8+ T cells play a potential dichotomous role in NAFLD progression and resolution.

**Figure 4:**
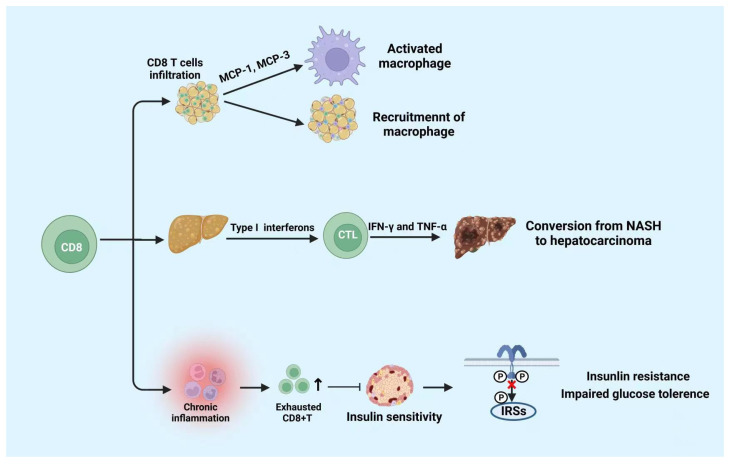
Functions of CD8+ T cells in metabolic diseases.

Nishimura et al. discovered that the accumulation of CD8+ T cells results in insulin resistance and chronic inflammation in mice [82]. Chronic inflammation can increase the amount of exhausted CD8+ T cells. The amount of exhausted CD8+ T cells and the pro-inflammatory cytokines produced by them are positively correlated with fasting blood glucose. Notably, the increase in CD8+ T cells accompanied by a senile phenotype in the peripheral blood and AT may accelerate the hyperglycemia in humans [14,95]. Moreover, the research also proclaimed that the exhausted CD8+ T cells impaired insulin sensitivity in both humans and mice [14,95]. Studies showed that in DIO, chemokines secreted by CD8+ T cells that infiltrate AT induce the activation of macrophages and facilitate the recruitment of macrophages into AT [82]. Overall, these researchers prove that CD8+ T cells promote the development of metabolic diseases through pro-inflammatory activities; Figure 4 summarizes it.

CD8+ T cells play a role in pro-inflammatory activities. CD8+ T cells infiltration promotes the activation and recruitment of macrophages. Moreover, CD8+ T cells infiltration in liver tissue is beneficial to the conversion from NASH to hepatocarcinoma. Moreover, cumulated exhausted CD8+ T cells suppress the insulin sensitivity which leads to insulin resistance and impaired glucose tolerance. NASH: non-alcoholic steatohepatitis. The figure was constructed with BioRender (https://biorender.com, accessed on 7 September 2022).

## 5. Signaling Pathway Regulates T Cells Involved in the Development of Metabolic Diseases

**Figure 5:**
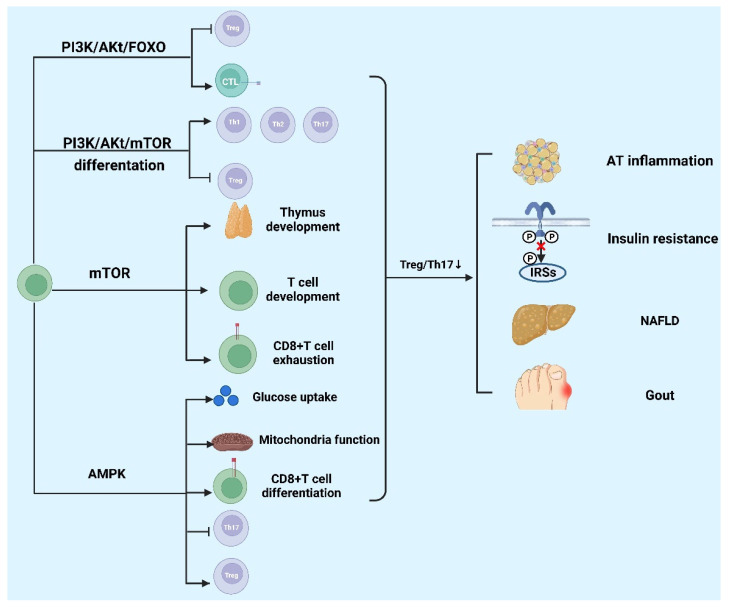
The signaling pathways regulating T cells in metabolic diseases. PI3K/Akt/mTOR signaling pathway promotes the differentiation of CD4+T cells. mTOR signaling pathway takes part in the development of thymus and T cells and the exhaustion of CD8+T cells. PI3K/Akt/FOXO suppresses the development of Tregs and promotes the expression of cytotoxicity effector. AMPK signaling pathway negatively regulates Th17 differentiation, glucose uptake, mitochondria function, and CD8+T cell differentiation, whereas the pathway positively regulates Treg cells’ differentiation. These pathways could regulate the ratio of Treg to Th17 together. The decrease in the ratio leads to AT inflammation, insulin resistance, NAFLD, and gout. PI3K: phosphatidylinositol 3-Kinase; Akt: protein kinase B; mTOR: mammalian target of rapamycin; AMPK: Adenosine 5‘-monophosphate (AMP)-activated protein kinase; NAFLD: non-alcoholic fatty liver disease. The figure was constructed with BioRender (https://biorender.com, accessed on 7 September 2022).

Metabolic diseases have a high incidence but there is still no effective treatment. The processes of different signaling pathways regulating T cells to participate in metabolic diseases are described in the following sections (Figure 5), which may provide directions for future treatment.

### 5.1. The mTOR Signaling Pathway

mTOR is an atypical serine/threonine protein kinase, which is relatively conservative in evolution. It can integrate nutrients, energy and growth factors, and other extracellular signals, participate in biological processes such as gene transcription, protein translation, ribosomal synthesis, and play an extremely important role in cell growth, apoptosis, autophagy, and metabolism [96]. The mammalian target of rapamycin (mTOR) is the central kinase of two protein complexes, mTORC1and mTORC2, which can be distinguished by their scaffold proteins, RAPTOR in mTORC1 and RICTOR in mTORC2, respectively [97]. mTOR plays a major role in the development, differentiation, and exhaustion of T cells. Firstly, the mTOR signaling supports the development of T cells. The deficiency of RAPTOR inhibits the cell cycle of DN1(a species of DN), suggesting that activated mTORC1 promotes the T cell development [98], whereas proliferation and glycolysis are significantly reduced in Sin1 (a component of mTORC2)-deficient DN thymocytes [99]. It also has been demonstrated that mTOR1 or mTOR2 inhibit the development of Treg cells [100,101]. Among them, mTOR signaling can further affect the development of the thymus. Hoshii et al. discovered that the thymus atrophied after removing all RAPTOR from tissues in mice [102]. Secondly, upon antigen recognition, CD4+ T cells differentiate into Th1, Th2, Th17, and Treg cells, and the differentiation processes are largely influenced by mTOR signaling. The development of Th1 depends on IL-12 and STAT4 activation. Delgoffe et al. discovered that the CD4+ T cells failed to activate GTPase Rheb to differentiate into Th1 cells due to mTOR1 deficiency and showed reduced STAT4 phosphorylation in response to IL-12 [103]. Th2 highly expresses transcription factor GATA3 and cytokines IL-4, IL-5, and IL-13. An experiment conducted by Delgoffe et al. showed that CD4+ T cells in mice lacking RAPTOR cannot produce IL-4 [104], indicating that the development of Th2 is highly related to mTOR2, not mTOR1. Shi et al. reported that HIF-1α was expressed in Th17 cells [105]. The Warburg effect indicates that mTORC1 can induce the expression of MYC and hypoxia-inducible factor (HIF), then mediate the production of glycolytic enzymes and transporters, and promote aerobic glycolysis [106]. In T cells lacking mTOR, glycolysis and Th17 development are suppressed, but the development of Treg was promoted by the inhibition of glycolysis. mTOR is expressed at low levels throughout the life cycle of Tregs, and Sauer et al. found that low culture condition can promote their development [107]. Finally, high lipids may cause T cells’ metabolic failure. Ma et al. stated that cholesteryl induces T cell failure by upregulating the expression of immune checkpoints on CD8+ T cells [108].

Moreover, pieces of evidence have shown that inhibition of the mTOR signaling pathway by some drugs can delay the development of metabolic diseases. Kamyshnyi found that patients with diabetes had an aggravated course of disease after COVID-19, while after taking metformin, the insulin sensitivity and blood glucose levels decreased, which further reduced the risk of Corona Virus Disease 2019 (COVID-19) [109]. This may be related to the inhibition of the mTOR pathway by metformin through the AMPK-dependent or independent mechanism [110]. Low expression of mTOR can reduce islet inflammation, promote Treg cell differentiation, and improve diabetes. Another experiment indicated that monocytes cocultured with metformin and MSU crystals in vitro had significantly lower mortality than monocytes cultured with MSU crystals alone, and the mTOR gene expression significantly reduced, which also indicates that metformin can inhibit the expression of mTOR, thereby reducing the death of monocytes and reducing inflammatory activity [111]. Clinical experiments have also shown that the incidence of gout is reduced after taking metformin [112,113]. Rapamycin can also effectively inhibit the mTOR signaling pathway, studies have shown that by feeding rapamycin to HFD-fed mice, Treg cells increases, insulin sensitivity decreases, and oxygen consumption increases [114].

mTOR is important in metabolic diseases. An increased proportion of Treg / Th17 can reduce AT inflammation and insulin resistance caused by obesity. In terms of NAFLD, the inflammation of liver can be reduced by alleviating liver inflammation and fibrosis, thereby inhibiting the progress of T2D and promoting the islets’ health, finally preventing inflammation caused by NAFLD.

### 5.2. The Phosphatidylinositol 3-Kinase (PI3K)/Protein Kinase B (Akt) Pathway

The PI3K/Akt signaling pathway is associated with proliferation, differentiation, and apoptosis, which is crucial in organismal growth and critical cellular processes by mediating growth factors [115]. Particularly, the IRS/PI3K/Akt signaling pathway is important for insulin to regulate the blood glucose balance [116].

PI3Ks are a family of lipid kinases [117]. PI3Ks contain three classes; due to the various activities, PI3K class I is the most deeply studied lipid kinase [118]. Akts contain three subtypes (Akt1, Akt2, and Akt3) due to the differences in serine/threonine residues. Akt1 is extensively expressed throughout the body, AKt2 is mainly expressed in tissues that are sensitive to insulin, such as AT, and liver, and Akt3 is generally expressed in the testis and brain [118,119].

The PI3K signal pathway is essential for native CD4 T cells to differentiate into the main Th cell subsets, Th1, Th2, Th17, and TFH [120,121,122]. During either thymocyte selection (natural Tregs; nTreg) or stimulation under tolerogenic conditions (iTreg), Tregs are generated [123]. PI3K blocked the clonal proliferation of CD4 T cells induced by suppressive antigens and APCs, in which p110δ plays a major role [124,125]. In addition, generous studies indicated that PI3K signaling and its downstream effectors also mediated CD4 T cell differentiation, particularly in the Akt/FOXO axis and TOR.

PI3K plays an important role in Th1 and Th2 differentiation through complex mechanisms. mTOR is a crucial signaling protein to connect PI3K activation and Th differentiation. In mouse T cells with mTOR knockout, clonal proliferation of mouse T cells was reduced and differentiation into Th1, Th2, or Th17 subsets was completely inhibited [103]. Conversely, in TOR-deficient T cells, the induced expression of forkhead box P3 (FoxP3) and the signature transcription factor that drives the Treg procedure are increased [103]. It has been reported that Th1 and Th17 differentiation was prevented by blocking the TORC1 activation [104].

Recent studies suggested that the development of nTregs is also regulated by PI3K-Akt- Forkhead Box O (FOXO). Moreover, it has been reported that the combined deletion of Foxo1 and Foxo3 can suppress the development of FoxP3+ nTregs in the thymus [126,127]. When FOXO factors bind to FOXO consensus sequences in the Foxp3 promoter, FoxP3 expression is activated [127,128]. However, PI3K generally inhibits the activity of FOXO. Therefore, FOXO factors can reposition the nucleus to initiate Tregs after PI3K activity is suppressed.

Additionally, it is reported that restriction of PI3K/Akt/TOR activity promotes CD4 T cells to convert to iTregs [123]. Other works support the role of PI3K/Akt signaling in counteracting iTregs conversion via suppressing FOXO activity [123]. iTregs induction is abrogated in T cells with Foxo1 and Foxo3 knockout, or in cells expressing active Akt [100,127,129].

Furthermore, activated CD8+ T cells require PI3K/Akt signaling to initiate the transcription function of CTL. Akt activity and FOXO inactivation are associated with the expression of cytotoxicity effectors, including perforin, granzymes, and IFN-γ [123]. AKT activation also regulates the balance of homing and trafficking receptors’ expression which allow cytotoxic T lymphocytes to move from lymph nodes to the sites of infection [123].

Evidence has shown that PD-1-deficient Treg cells can inhibit PI3K/Akt signaling, thereby enhancing Treg immunosuppressive ability, and in mice that only have a PD-1 deficient in Tregs, their diabetes has improved [130]. Together, the above conclusions show that the PI3K/Akt pathway is important in the differentiation of T lymphocytes. Differentiated T lymphocytes are involved in the inflammation response of tissues. In obesity-induced chronic inflammation, adipokines and adipose hypertrophy lead to insulin resistance through inhibiting PI3K/AKT signaling which suppresses lipolysis, glucose utilization (GU), and the ability of sterol regulatory element binding protein (SREBP) to boost lipogenesis [131]. Restriction of the PI3K/Akt pathway is beneficial to promote the differentiation of Treg and suppress the differentiation of Th1 and CD8+ T cells, thereby attenuating the inflammation response in metabolic diseases. Therefore, the inhibitor of PI3K/Akt pathway may be a potential treatment for metabolic diseases.

### 5.3. The AMP-Activated Protein Kinase (AMPK) Pathway

AMPK is an AMP-activated protein kinase, which is a key molecule in the regulation of bioenergy metabolism, and plays an indispensable role in T cell metabolism and function [132]. In the mTOR signaling pathway, the upstream of AMPK is regulated by LKB1 and CaMKK2, which activate AMPK under low energy metabolic stress and Ca^2+^ respectively, while the tumor suppressor gene FLCN inhibits activation of AMPK, phosphorylation of TSC2 (activation) and RAPTOR (inhibition) downstream of AMPK inhibit mTOR activity, which in turn reduces mRNA translation [133]. The catalytic α subunit of AMPK is encoded by two genes, Prkaα1 and Prkaα2, but in mouse T cells, Only the α1 catalytic subunit is expressed [134].

A function of AMPK towards T cells is regulating fuel usage, as activated T cells possess glucose-sensitive metabolic checkpoints controlled by the energy sensor AMPK, which regulates messenger RNA (mRNA) translation as well as mitochondrial metabolism. Blagih et al. noted that the capacity of GU and the function of mitochondria were reduced in AMPKα1-deficient T cells, which leads to decreased effector T cell responses to pathogens [135]. Pearce et al. concluded that mice with cell-specific depletion of TRAF6 have defects in producing CD8+ memory T cells, while the antidiabetic drug metformin, which acts as an AMPK agonist, restored the memory CD8+ T cells production [136]. It is reported that the AMPK-deficient effector T cells are more sensitive to apoptosis in the inflammatory environment in vivo [135], implying that effector T cells cannot survive in the inflammatory environment. Moreover, recent researchers have shown that AMPK plays a part in the differentiation of T cells under the asymmetric inheritance of mTORC1. When T cells are activated, CD8 molecules differentiate into progeny cells. That is, CD8hi cells in the proximal part of APC differentiate into effector T cells with high mTORC1 and low AMPK levels. Conversely, CD8lo cells in the distal part of APC differentiate into memory T cells with low mTORC1 and high AMPK levels [137,138]. For CD4+ T cells, CD4 can differentiate into Th17 cells in the presence of a fatty acid synthesis enzyme, such as Acetyl-CoA Carboxylase (ACC). However, lower levels of ACC activity contribute to the differentiation of Treg cells [133]. AMPK, as a direct inhibitor of ACC, will affect the balance between Th17 and Treg. Regarding metabolic diseases, the AMPK signaling pathway mainly acts on CD8+ T cells. Under low levels of AMPK, an increase in effector CD8+ T cells is indispensable in the initiation and spread of adipose inflammation [82]. Finally, the imbalance of Th17/Treg also affects the progression of T2D, hyperuricemia, and other diseases.

Also, research carried by Nyambuya showed that metformin has anti-inflammatory effects by activating the AMPK signaling pathway and inhibiting the mTOR signaling pathway, thereby slowing down the progression of T2D and T2D-related cardiovascular complications [139].

## 6. Conclusions

Metabolic diseases are common worldwide; popular examples include obesity and NAFLD. Inflammation and oxidative stress are the main pathogenesis of metabolic diseases, and chronic or excessive inflammation can affect their progression, and T cells play an indispensable role in inflammation. In this review, we focused on the role of T cells in regulating inflammation in different metabolic diseases and the role of signaling pathways in regulating T cells, as well as the latest research progress and future direction. In metabolic diseases, such as obesity, NAFLD andT2D, CD4+ T cell differentiated cells, such as Th1, Th2, Th17, and Tregs, all play potential roles in disease regulation or progression. However, there are still several mechanisms worth studying. The functions of Th2 cells in metabolic diseases are not well understood [64], and the mechanism of Th1 cells and the role of Th2-related cytokines also need further research in gout [140]. Overall, the function of T cells in metabolic diseases is thought-provoking. It is also worth exploring whether immunotherapy may become an original way for the treatment of metabolic diseases as many trials have been conducted to demonstrate the effectiveness of immunotherapy. Imbalance in cytokine levels is an important feature of metabolic diseases, and cytokine-specific monoclonal antibody therapy can reduce disease progression [141]. A phase I trial of an IL-1 antibody in diabetic patients has shown initial results [142]. In addition, by inhibiting the homing of macrophages to AT, it can also improve insulin resistance in obese mice [143]. Moreover, the AT inflammation is associated with NK cells, T cells, and B cells [141]. By reducing the expression of INF-γ (a product of T cells and NK cells), systemic inflammation has been reduced and insulin resistance has been improved [144]. Moreover, different signaling pathways are also involved in the development, differentiation, and exhaustion of T cells. However, there are still several issues that remain to be considered: (a) How to eliminate the adverse side effects caused by completely blocking mTOR signal? (b) How do different PI3K inhibitors impact the differentiation and transport of pathogenic T lymphocytes? (c) Whether the inhibitors of PI3K downstream effectors could become the potential medicine for metabolic diseases? (d) How to improve the use of direct AMPK activators in vivo?

Lastly, translating our knowledge of these signaling pathways in T cells into therapeutic modalities remains a challenge in the future. Future studies on the regulation of T cells by numbers of signaling pathways will facilitate the development of targeted immunotherapies for multiple metabolic diseases.

## Data Availability

Not applicable.
